# NeuroFusion: A Unified Framework for Generalized Visual Stimulus Decoding from fMRI Across Datasets and Subjects

**DOI:** 10.1007/s12021-026-09803-3

**Published:** 2026-07-18

**Authors:** Muhammad Kashif, Matteo Ferrante, Nicola Toschi

**Affiliations:** 1 Department of Biomedicine and Prevention, University of Rome Tor Vergata, Rome, Italy; 2 Martinos Center for Biomedical Imaging, MGH and Harvard Medical School, Boston, MA USA

**Keywords:** fMRI decoding, Cross-subject generalization, Cross-dataset transfer, Contrastive learning, Diffusion models, IP-Adapter, Stable Diffusion XL, Visual reconstruction

## Abstract

Recent advancements in neural decoding have shown promising results in reconstructing visual experiences from brain activity. However, existing approaches focus primarily on decoding within a single dataset or subject, which limits generalization across various sources of neuroimaging. In this work, we propose a novel framework for the decoding of visual stimuli between subjects and between data sets, integrating neural recordings from multiple publicly available fMRI datasets. To address inherent intersubject and interdataset variability, we introduce a contrastive learning-based alignment strategy using image embeddings from a pre-trained IP-Adapter model. Our approach learns a shared latent space by aligning subject-specific neural representations with image features, enabling generalized decoding across both subjects and datasets. In addition, we propose a simple yet effective data augmentation method using ridge regression. This method synthesizes realistic fMRI-like signals from novel images by predicting voxel activity and injecting learned noise distributions, thus enhancing training diversity and model robustness. To the best of our knowledge, while several recent studies have explored cross-subject decoding, we extend recent cross-subject decoding efforts by training a single unified framework jointly across multiple public fMRI datasets and subjects, enabling cross-dataset transfer in addition to cross-subject generalization. We distinguish this multi-dataset unified training setting, where each dataset contributes training data, from a stricter leave-one-dataset-out transfer setting in which the target dataset is excluded from source pretraining and used only for lightweight alignment-layer adaptation. Empirically, our unified model achieves strong semantic reconstruction across datasets (e.g., up to 94.8% CLIP similarity on NSD (AUG) and 0.403 SSIM on BOLD5000 after lightweight finetuning), demonstrating robust cross-subject and cross-dataset transfer.

## Introduction

Human perception involves the integration of diverse sensory inputs such as vision, audition, olfaction, and touch, which are processed through complex and distributed neural computations (Xia et al., 2018; Yeung et al., 2024). Among these, visual stimuli play a central role in how we interpret and respond to the external world. However, visual information is not mapped to brain activity in a direct or linear fashion (Kasteleijn-Nolst Trenité et al., 2004; De Cheveigné et al., 2018). Instead, stimuli are encoded through high-dimensional neural patterns shaped by cognition, attention, and individual variability (Kriegeskorte & Douglas, 2018). This complexity has motivated the field of brain decoding, which seeks to reconstruct or interpret external stimuli from neural activity, offering insights into mental representation and perception (Haynes, 2009; Tong & Pratte, 2012; Saeidi et al., 2021).

Functional magnetic resonance imaging (fMRI) is widely used in brain decoding due to its high spatial resolution (typically 1–2 mm voxel size) and ability to capture whole-brain activity in a non-invasive and safe manner (Goense et al., 2016), making it well-suited for cognitive neuroscience research. fMRI measures blood-oxygen-level-dependent (BOLD) signals, which are indirect indicators of neural activity. While spatially detailed, fMRI suffers from relatively low temporal resolution due to the repetition time (TR) and the sluggish nature of the hemodynamic response function (Logothetis, 2008; Huettel et al., 2004).

Recent advances in deep learning, including generative adversarial networks (GANs) (Ozcelik et al., 2022; Shen et al., 2019) and diffusion models (Ozcelik et al., 2022; Scotti et al., 2023; Takagi & Nishimoto, 2023), have significantly improved the realism and semantic fidelity of reconstructed images from fMRI data. These approaches typically learn a mapping from brain signals to image embeddings or pixel-level reconstructions, enabling end-to-end models for perceptual decoding (Naselaris et al., 2011; Kamitani & Tong, 2005; Haynes & Rees, 2006). However, most prior work in brain decoding has focused on subject-specific settings, where models are trained and evaluated on data from the same individual (Gu et al., 2022; Mai & Zhang, 2023; Ozcelik & VanRullen, 2023; Scotti et al., 2023; Takagi & Nishimoto, 2023). This limits scalability and generalization, as each new subject requires retraining or adaptation. More recently, cross-subject decoding has emerged as a promising direction, where models are trained on data from multiple individuals and tested on new subjects (Xia et al., 2024; Lin et al., 2022; Wang et al., 2024b; Chen et al., 2023; Ferrante et al., 2024a). These efforts aim to learn representations that generalize across individual anatomical and functional differences, moving toward more universal decoders. Yet, an important gap remains: all cross-subject models are confined to a single dataset, assuming consistent acquisition parameters, stimulus paradigms, and preprocessing pipelines. In contrast, practical applications would benefit from decoders that not only generalize across subjects but also across datasets collected under heterogeneous conditions. This setting cross-dataset and cross-subject decoding poses additional challenges due to domain shift, differing voxel resolutions, and varying visual distributions, but it also brings the field closer to practical, robust neural decoding systems.

In this context, cross-subject and cross-dataset brain decoding represents an important extension of previous efforts toward building more generalizable neural representations of perception. Cross-subject and cross-dataset decoding provides a stringent test of whether shared semantic representations of vision can be recovered from fMRI despite variability in subjects, scanners, and stimulus sets. By jointly training across heterogeneous datasets, we test the extent to which a common representational geometry supports generalization. This setting also increases training diversity and reduces the need for per-dataset model redesign. In the present work, “multi-dataset unified training” refers to training one shared model on the training splits of NSD, BOLD5000, and GOD, whereas “leave-one-dataset-out” (LODO) refers to excluding the target dataset from source pretraining and using the target training split only for alignment-layer adaptation before evaluation on the target test split.

In this study, we propose a unified decoding model that leverages vision-language semantic embeddings derived from Stable Diffusion XL (SDXL) (Podell et al., 2023) and IP-Adapter (Ye et al., 2023). SDXL is a high-capacity latent diffusion model designed for high-resolution image generation; it operates by iteratively denoising latent representations to synthesize semantically coherent images, conditioned on textual or visual prompts (Rombach et al., 2022; Podell et al., 2023). To personalize and adapt these generative models to individual embeddings, we utilize IP-Adapter (Ye et al., 2023), a lightweight plug-in mechanism that injects image embeddings (e.g., from CLIP) into the cross-attention layers of diffusion models without fine-tuning the entire model. These components project fMRI activity into a shared latent space of semantic concepts, allowing flexible and interpretable alignment between brain signals and visual representations. Our approach advances scalable and cognitively meaningful brain decoding by enabling robust cross-domain generalization and providing new tools to study visual perception at the systems neuroscience level, while also supporting real-world applications in brain–computer interfaces and neural diagnostics.

Specifically, (i) We introduce a unified framework for cross-subject and cross-dataset fMRI-to-image decoding by aligning neural activity to IP-Adapter visual embeddings using contrastive learning. (ii) We propose subject-/dataset-specific alignment modules that map heterogeneous voxel spaces into a shared latent space used by a shared decoder. (iii) We present a ridge-regression-based augmentation procedure that synthesizes subject-specific fMRI-like samples for novel images via residual-noise modeling. (iv) We evaluate cross-subject and cross-dataset generalization across NSD, BOLD5000, and GOD, including transfer with lightweight finetuning. We additionally report a LODO transfer experiment to separate joint multi-dataset training from transfer to a dataset that was not used during source pretraining.

## Related Work

Decoding distributed patterns of brain activity to infer external stimuli—often termed brain decoding—has become a central goal in cognitive neuroscienc (Naselaris et al., 2011; Shirakawa et al., 2024; Shen et al., 2019). Functional magnetic resonance imaging (fMRI), due to its non-invasive nature and full-brain coverage, is widely used for studying the neural basis of perception, especially in visual paradigms (Amaro & Barker, 2006; Engel et al., 1994). Traditional decoding pipelines generally follow two stages: mapping fMRI signals to high-dimensional image features extracted from pretrained visual models, then reconstructing stimuli via generative models (Shen et al., 2019; Beliy et al., 2019; Lin et al., 2022). Early work focused on decoding low-level features like orientation, color, or object categories (Kamitani & Tong, 2005; Miyawaki et al., 2008), though these approaches were limited by fixed vocabularies. The rise of deep learning particularly CLIP, GANs, and diffusion models enabled prediction of rich semantic embeddings and realistic image reconstructions from brain data (Nishimoto et al., 2011; Chen et al., 2024; Fang et al., 2023; Lu et al., 2023). Diffusion models iteratively denoise latent noise using learned priors, where brain-derived representations serve as conditioning inputs to produce coherent reconstructions (Lu et al., 2023; Chen et al., 2024). However, most models are trained in a subject-specific manner, limiting their generalizability. To address inter-subject variability, studies have proposed anatomical normalization, functional alignment such as hyperalignment (Haxby et al., 2011), and joint models with shared encoders and subject-specific parameters (Ruffle et al., 2024). Contrastive learning has also been applied to align image embeddings with corresponding neural activations across subjects (Wang et al., 2024a; Ferrante et al., n.d.), though mostly within single datasets. Cross-dataset decoding remains less systematically studied than within-dataset and cross-subject decoding, and reported evaluations often differ in preprocessing, voxel selection, and stimulus distributions, making transfer claims difficult to compare directly.

While prior work in brain decoding has made significant strides in subject-specific and cross-subject generalization, several recent approaches particularly those based on hyperalignment (Zhang et al., 2025) and multimodal embedding strategies (Rajabi et al., 2025; Wang et al., 2024a) have demonstrated improved transferability across subjects and tasks. These alignment techniques, which match voxel-wise functional topographies or jointly embed brain and visual features, offer complementary benefits to contrastive methods. A deeper comparison with such baselines, including performance on cross-dataset settings, is a promising direction for future work and could contextualize the benefits of the proposed approach.

To our knowledge, no prior work has systematically addressed decoding across entirely different datasets, which is essential for building transferable systems. This challenge is timely, given the success of foundation models that generalize across domains via diverse training data (Radford et al., 2021; Zhuang et al., 2020). In this work, we take a first step toward bridging this gap by proposing a unified model for both cross-subject and cross-dataset brain decoding. Our hybrid architecture uses shared components to capture visual-semantic representations, along with subject- and dataset-specific adaptation layers. We apply contrastive learning across datasets to align fMRI signals with shared image embeddings, grounding them in a common semantic space despite variability in subjects and acquisition. This promotes robustness, generalization, and scalability toward universal brain decoding systems.

## Methods

**Fig. 1:**
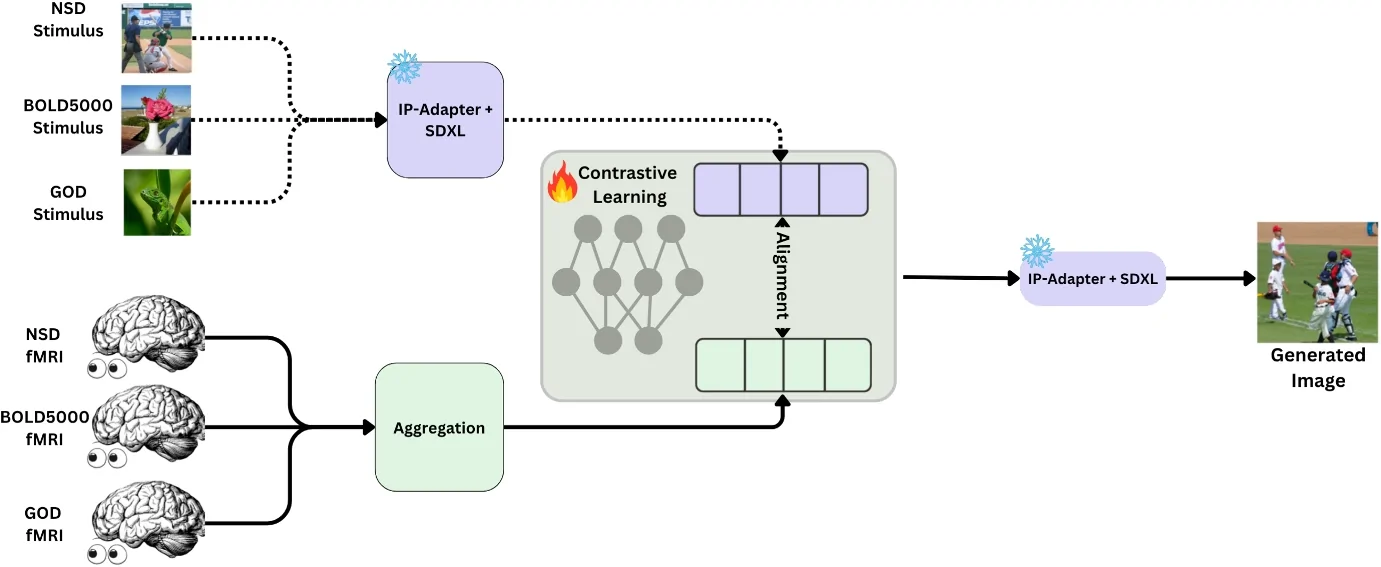
Overview of the proposed method. Stimuli from NSD, BOLD5000, and GOD datasets are encoded using the IP-Adapter image encoder (OpenCLIP backbone) to extract image embeddings; these embeddings are later injected via IP-Adapter to condition SDXL during reconstruction. Corresponding fMRI signals are aggregated into a unified training corpus by pooling all (fMRI, subject-id) tuples from NSD + BOLD5000 + GOD into one big dataset and aligned to these embeddings using a contrastive learning framework. The estimated embeddings are then used to generate images via the IP-Adapter + SDXL pipeline

Our objective is to develop a generalized brain decoding framework that can effectively reconstruct visual stimuli from fMRI signals across different subjects and datasets. To achieve this, we propose a modular pipeline that leverages large-scale vision-language models, contrastive representation alignment, and generative image synthesis. The overall framework is illustrated in Fig. 1.

### Overview

The proposed model decodes brain activity into semantic visual representations by aligning neural signals with high-dimensional image embeddings. Specifically, we utilize the image encoder from the IP-Adapter (Ye et al., 2023) for stable diffusion XL (SDXL) 1.0 (Podell et al., 2023), instantiated from the pretrained checkpoint ”stabilityai/stable-diffusion-xl-base-1.0” with the IP-Adapter weights ”h94/IP-Adapter” (sdxl-models/ip-adapter-sdxl.bin). The visual backbone is the OpenCLIP ViT-bigG-14 encoder (OpenCLIP implementation), which outputs a 1280-dimensional global image embedding (corresponding to the projected [CLS] representation; embedding size = 1280). This model is a large-scale vision transformer with approximately 1.8 billion parameters, trained on LAION-2B and other large web-scale datasets (Cherti et al., 2023). It produces global image embeddings from the [CLS] token of the final transformer layer, without including projected patch tokens, following the standard OpenCLIP implementation. These embeddings serve as the target space for neural representation learning. Given an fMRI sample $x_{\textrm{fMRI}}$ from a subject observing an image *I*, our model learns a mapping function $f_n(\cdot )$ that transforms the brain signal into a latent vector $z_{\textrm{fMRI}} = f_n(x_{\textrm{fMRI}})$. The corresponding image embedding is denoted by $z_{\textrm{IP}} = h(I)$, where *h(· )* is the IP-Adapter encoder. To align these modalities, we use a contrastive learning strategy that minimizes a symmetric cross-entropy loss over the cosine similarities between the projected neural and image embeddings (see Section “Cross-subject and Cross-dataset Neural Vision Alignment”).

We distinguish two cross-dataset protocols. In the multi-dataset unified protocol, the shared encoder is trained jointly on the training splits of NSD, BOLD5000, and GOD, and each dataset is evaluated only on its held-out test split. Thus, the target dataset is included during training, but its test stimuli are never used for training or model selection. In the LODO protocol, the target dataset is excluded from source pretraining; the source model is trained on the other two datasets, then the shared encoder is frozen and only the target subject-specific alignment layers and final alignment layer are finetuned using the target training split before evaluation on the target test split.

### Datasets

To evaluate our approach for brain decoding across subjects and datasets, we utilized three publicly available fMRI datasets that are widely recognized in the field of visual neuroscience: the Natural Scenes Dataset (NSD) (Allen et al., 2022), BOLD5000 (Chang et al., 2019), and Generic Object Decoding (GOD) (Horikawa & Kamitani, 2017). These datasets differ in several critical aspects that introduce inter-dataset variability. NSD was collected using a 7T MRI scanner, whereas BOLD5000 and GOD were acquired on 3T scanners. The visual stimuli also vary: NSD includes natural scenes, BOLD5000 comprises a wide range of everyday objects and scenes, and GOD focuses on generic object categories. Additionally, the datasets differ in subject pool sizes and stimulus presentation protocols. This diversity allows us to assess the generalizability of our framework under varied experimental conditions. A summary of all datasets is provided in Table 1.

#### Natural Scenes Dataset (NSD)

The NSD offers high-resolution fMRI recordings from eight participants who were shown a large set of naturalistic images drawn from the COCO image collection (Xia et al., 2024). Each image was displayed for three seconds, followed by a one-second inter-stimulus interval. We used NSD subjects 1, 2, 5, and 7. For each participant the training set included approximately 8,859 images and 24,980 corresponding fMRI trials, while the testing set comprised 982 images and 2,770 trials. We used the provided GLM beta estimates (single-trial betas) and z-scored voxel responses within each subject using training-set statistics. Voxels were selected using NSD ROI masks restricted to early visual areas (V1, V2, V3, V4) and higher-level ventral and dorsal visual regions (EBA, FFA-1, FFA-2, OFA, PPA, OPA, RSC, VWFA-1, VWFA-2), collectively defined by the NSD nsdgeneral mask, resulting in approximately 15,000 voxels per subject.

#### BOLD5000

The BOLD5000 dataset consists of fMRI data collected while participants viewed a broad range of natural and object-centric images, including samples from COCO, ImageNet, and SUN. It comprises 4,916 unique stimuli, with 4,803 of them shown once and 113 presented multiple times, yielding a total of 5,254 trials. Data were recorded from four individuals (CSI1–CSI4), three of whom completed the entire set of trials. Each image was presented for one second, followed by a nine-second fixation period. Data were acquired using a 3T Siemens Prisma scanner with a gradient-echo EPI sequence (TR = 2000 ms, TE = 30 ms, flip angle = 75°, voxel size = 2 mm isotropic). We adopted a standardised train-test split from Chen et al. (2023): all unique-image trials (4,803 samples) were used for training, while trials corresponding to repeated images (113 samples) were used for evaluation. We use the released GLM beta-weight representations provided in the BOLD5000 preprocessing pipeline and restrict analysis to visual cortex voxels, resulting in approximately 2,500 voxels per subject after masking. Voxels are z-scored per subject using training-set statistics.

#### Generic Object Decoding (GOD)

The GOD dataset contains fMRI measurements from five individuals engaged in either image perception or imagery tasks involving objects from the ImageNet database. We use only the perception condition (image viewing) for all GOD experiments. During the training phase, subjects viewed 1,200 images spanning 150 object categories, with each category represented by eight unique images. The testing phase included 50 distinct object categories, each represented by a single image repeated across 35 trials. Stimuli were shown for nine seconds per trial. Recordings were organized into discrete runs — 24 for training and 35 for testing — acquired using a 3T scanner with an EPI sequence (TR = 3000 ms, TE = 30 ms, voxel size = 3 mm$^{3}$). We extract visual cortex voxels ( 4,500 per subject) and compute responses as the average of TRs 3–6 post-stimulus onset (corresponding to 6–16 s after stimulus onset), following the dataset’s recommended protocol. Data preprocessing included motion correction, detrending, and spatial normalization.

**Table 1:** Summary of the three fMRI datasets used in this study. “Subjects used” refers to the subset selected for our experiments. Voxel counts are approximate and vary slightly per subject

Dataset	Scanner	Subjects (used)	Train trials	Test images	Voxels/subject	TR (ms)	Stim. duration	Image source
NSD (Allen et al., 2022)	7T Siemens	8 (4)	*∼ *24,980	982	*∼ *15,000	1600	3 s	COCO
BOLD5000 (Chang et al., 2019)	3T Siemens	4 (4)	4,803	113	*∼ *2,500	2000	1 s	COCO, ImageNet, SUN
GOD (Horikawa & Kamitani, 2017)	3T	5 (5)	1,200	50	*∼ *4,500	3000	9 s	ImageNet

### Cross-subject and Cross-dataset Neural Vision Alignment

In this section, we describe our core contribution, how we train the model across several datasets. We introduced a contrastive alignment framework which aim is to map fMRI signals into a shared latent space where they can be meaningfully aligned with image embeddings obtained from the pretrained IP-Adapter encoder (Ye et al., 2023). This alignment enables effective visual stimulus decoding across multiple datasets and individuals. Let *h(· )* denote the pretrained IP-Adapter image encoder, which maps an input image *I* to its semantic representation $z_{\textrm{IP}} = h(I) \in \mathbb {R}^d$. Given fMRI data $x_{\textrm{fMRI}} \in \mathbb {R}^V$, where *V* is the number of voxels, we define a neural encoder $f_n(\cdot )$ that predicts a corresponding neural representation $z_{\textrm{fMRI}} = f_n(x_{\textrm{fMRI}}) \in \mathbb {R}^d$, such that $z_{\textrm{fMRI}} \approx z_{\textrm{IP}}$ when the neural data $x_{\textrm{fMRI}}$ was recorded in response to image *I*. To handle inter-subject variability and align neural data from different sources, we structure the function $f_n(\cdot )$ as a composite mapping:

1 $$\begin{aligned} f_n(x_{\textrm{fMRI}}, s) = g_n(a_n(x_{\textrm{fMRI}}, s)) \end{aligned}$$

where $a_n(\cdot , s)$ is a subject and dataset specific alignment module that projects neural data from subject *s* into a common intermediate representation space, and $g_n(\cdot )$ is a shared neural decoder that maps this aligned space to the joint semantic space of visual embeddings. To preserve the semantic structure of the visual domain and avoid collapsing to trivial solutions, we adopt a contrastive learning strategy. For each sample in a batch, we compute both fMRI-derived and image-derived embeddings, and normalize them to lie on the unit hypersphere:

2 $$\begin{aligned} \tilde{z}_{\textrm{fMRI}, i} = \frac{z_{\textrm{fMRI}, i}}{\Vert z_{\textrm{fMRI}, i} \Vert _2}, \quad \tilde{z}_{\textrm{IP}, i} = \frac{z_{\textrm{IP}, i}}{\Vert z_{\textrm{IP}, i} \Vert _2} \end{aligned}$$

We then compute the similarity matrix between all pairs of fMRI and image embeddings in the batch:

3 $$\begin{aligned} \textrm{logits} = \frac{\tilde{z}_{\textrm{fMRI}} \cdot \tilde{z}_{\textrm{IP}}^\top }{\tau } \end{aligned}$$

Where *τ* is a temperature scaling factor that controls the sharpness of the similarity distribution. We define the contrastive loss using a symmetric cross-entropy objective, encouraging the correct neural-image pairs to have the highest similarity:

4 $$\begin{aligned} \mathcal {L} = \frac{1}{2} \left( \mathcal {L}_{\textrm{CE}}(\textrm{logits}, \textbf{t}) + \mathcal {L}_{\textrm{CE}}(\textrm{logits}^\top , \textbf{t}) \right) \end{aligned}$$

Here, $\textbf{t} = [0, 1, 2, \ldots , N-1]$ is the ground-truth label sequence for a batch of size *N*, indicating the correct matching pairs. The loss ensures that representations from the same stimulus (image and neural response) are pulled together in the embedding space, while mismatched pairs are pushed apart. Through optimization of this contrastive objective, our model learns a shared latent space where semantic visual and neural representations are aligned across datasets and subjects, thereby enabling robust generalization for brain decoding tasks.

### Data Augmentation

A major challenge in brain decoding is the limited availability of fMRI data, which hinders model generalization, especially across subjects and datasets. To address this, we introduce a principled data augmentation strategy that synthesizes biologically plausible brain responses for unseen visual stimuli, tailored per subject. We trained subject-specific encoding models that map image embeddings to fMRI voxel activity using ridge regression and controlled noise perturbation. Let $\textbf{Z}_{\textrm{IP}}^{(s)} \in \mathbb {R}^{N \times d}$ represent the image embeddings from the IP-Adapter and $\textbf{X}_{\textrm{fMRI}}^{(s)} \in \mathbb {R}^{N \times V}$ be the corresponding fMRI signals for subject *s*, where *d* denotes the embedding dimensionality and *V* the number of voxels. For each subject, we trained a separate ridge regression model to predict voxel activity from visual embeddings by minimizing the following objective:

5 $$\begin{aligned} \textbf{W}^{(s)} = \arg \min _{\textbf{W}} \left\| \textbf{X}_{\textrm{fMRI}}^{(s)} - \textbf{Z}_{\textrm{IP}}^{(s)} \textbf{W} \right\| _2^2 + \lambda \Vert \textbf{W} \Vert _2^2 \end{aligned}$$

where *λ* is a regularization hyperparameter. The subject-specific encoder is then defined as:

6 $$\begin{aligned} \hat{x}_{\textrm{fMRI}} = k_s(z_{\textrm{IP}}) = z_{\textrm{IP}} \textbf{W}^{(s)} \end{aligned}$$

which projects an image embedding $z_{\textrm{IP}}$ into the fMRI voxel space. Using this model, we synthesized new neural responses by applying $k_s(\cdot )$ to embeddings of novel images. These images were sampled from the ImageNet dataset and encoded using the pretrained IP-Adapter to obtain their latent representations $z_{\textrm{IP}}$. We sampled 8,859 images per subject to introduce additional variability and to simulate responses to unseen stimuli. To prevent train–test contamination, we remove any augmentation images that overlap with stimulus sets in NSD/BOLD5000/GOD (using available image identifiers and/or perceptual hashes). All augmentation images are therefore disjoint from evaluation stimuli. The predicted brain activity for each novel image was estimated as $\widehat{x}_{\textrm{fMRI}} = k_s(z_{\textrm{IP}})$. To model inter-subject and trial variability, we injected stochastic noise sampled from an empirical distribution of decoding residuals. Specifically, we first computed the squared residuals per voxel:

7 $$\begin{aligned} e = \left( \textbf{X}_{\textrm{fMRI}}^{(s)} - k_s(\textbf{Z}_{\textrm{IP}}^{(s)}) \right) ^2 \end{aligned}$$

where the squared residuals are computed per voxel and averaged over training samples, yielding a vector $e \in \mathbb {R}^{V}$ of per-voxel mean squared prediction errors.

To model the noise distribution, we fit a piecewise-linear continuous distribution to the empirical values $\{e_v\}_{v=1}^{V}$ using a 5-bin density histogram converted to a continuous distribution. Thiscaptures the heavy-tailed, non-Gaussian structure of residual magnitudes across voxels without assuming a parametric form. During synthesis, noise is sampled *independently per voxel and per augmented sample*:

8 $$\begin{aligned} \epsilon _{iv} \overset{\text {i.i.d.}}{\sim } \mathcal {H}(e), \quad i = 1,\ldots ,N_{\text {aug}},\; v = 1,\ldots ,V \end{aligned}$$

yielding a noise matrix $\boldsymbol{\epsilon } \in \mathbb {R}^{N_{\text {aug}} \times V}$. The final augmented brain response for subject *s* is then given by:

9 $$\begin{aligned} \hat{x}_{\textrm{fMRI}} = k_s(z_{\textrm{IP}}) + \sigma \cdot \boldsymbol{\epsilon }, \qquad \boldsymbol{\epsilon }_{iv} \overset{\text {i.i.d.}}{\sim } \mathcal {H}(e) \end{aligned}$$

where *σ* is a dimensionless noise scale factor: *σ = 1* injects noise whose magnitude is drawn from the same distribution as the per-voxel squared prediction error, while larger values of *σ* proportionally amplify the injected noise relative to the typical residual magnitude of the encoding model. Since fMRI signals are in raw (unnormalised) voxel-amplitude units at augmentation time and are subsequently z-scored per subject during training, the effective noise level after normalisation scales as $\sigma \cdot \sqrt{\bar{e}}\,/\,\hat{\sigma }_v$, where $\bar{e}$ is the mean per-voxel MSE and $\hat{\sigma }_v$ is the per-voxel training standard deviation.

We explored a grid of augmentation configurations with varying augmentation percentages $\in \{25\%, 50\%, 75\%, 100\%\}$ and noise scale factors $\sigma \in \{10, 15, 50, 75, 100\}$, generating distinct synthetic datasets for each combination. This augmentation strategy produced a large pool of subject-specific, stimulus-driven fMRI samples, allowing our decoding models to train on a richer and more diverse neural distribution. As a result, we observed improved robustness in cross-subject and cross-dataset decoding tasks. Our approach highlights the utility of encoder-based augmentation to overcome data limitations in brain decoding.

### Visual Reconstruction via Ridge-refined Embeddings and IP-Adapter-SDXL

To enable the reconstruction of visual stimuli from brain activity, we adopt a two-stage decoding pipeline. First, the predicted latent representations $z_{\textrm{fMRI}}$ from the neural encoder are further refined using a linear regression model to enhance their alignment with the visual latent space of the IP-Adapter. Specifically, we employ ridge regression, a regularized linear model that mitigates overfitting and captures stable mappings from fMRI-derived features to semantic image embeddings. This refinement step serves a concrete purpose: the neural encoder is optimised via a contrastive objective that encourages directional alignment between predicted and target embeddings, but does not explicitly minimise the residual linear bias between the two spaces. Ridge regression corrects this systematic offset by learning a linear map from predicted to target embeddings in a least-squares sense, with $\ell _2$ regularisation to prevent overfitting. Crucially, the refinement model $\mathcal {R}$ is trained exclusively on training-set predictions and their corresponding ground-truth embeddings, with no access to test stimuli, ensuring that no test-set information leaks into the reconstruction pipeline. Let $z_{\textrm{fMRI}} \in \mathbb {R}^d$ denote the output of the neural encoder for a given fMRI input. We fit a subject-agnostic ridge regression model $R: \mathbb {R}^d \rightarrow \mathbb {R}^d$, trained to map $z_{\textrm{fMRI}}$ to the ground-truth image embeddings $z_{\textrm{IP}}$, minimizing the following objective:

10 $$\begin{aligned} W_R = \arg \min _{W} \Vert Z_{\textrm{IP}} - Z_{\textrm{fMRI}} W \Vert _2^2 + \lambda \Vert W \Vert _2^2 \end{aligned}$$

where $W_R \in \mathbb {R}^{d \times d}$ is the ridge regression weight matrix. The refined embedding is then given by:

11 $$\begin{aligned} z_{\textrm{reg}} = R(z_{\textrm{fMRI}}) = z_{\textrm{fMRI}} W_R \end{aligned}$$

The refined embeddings $z_{\textrm{reg}}$ are then fed into the IP-Adapter conditioned on Stable Diffusion XL (SDXL) (Podell et al., 2023) to reconstruct visual scenes. We use the publicly available stabilityai/stable-diffusion-xl-base-1.0 checkpoint with the ip-adapter_sdxl.bin weights from the h94/IP-Adapter, loaded via the diffusers library. The IP-Adapter scale is set to 0.6. Concretely, the refined embedding $z_{\textrm{reg}} \in \mathbb {R}^{1 \times 1280}$ is structured into a two-element prompt tuple $(z_{\textrm{uncond}}, z_{\textrm{reg}})$, where $z_{\textrm{uncond}} = \textbf{0}$ is the unconditional (null) embedding used for classifier-free guidance, following the standard IP-Adapter inference protocol. Image generation uses 50 denoising steps with an empty text prompt (prompt = “ ”) and a fixed negative prompt of *“deformed, ugly, wrong proportion, low res, bad anatomy, worst quality, low quality”* to suppress low-quality outputs. All images are generated with a fixed random seed of 33 to ensure reproducibility across subjects and evaluation runs. All computations are performed in float16 precision on an NVIDIA H100 GPU. This decoding strategy leverages the representational richness of SDXL while maintaining high-level semantic fidelity from brain-derived embeddings, resulting in coherent and visually plausible reconstructions.

## Results

All experiments were implemented using Python 3.12 with the PyTorch Lightning framework. Training was conducted on an NVIDIA H100 GPU 80GB, leveraging its high-throughput capabilities for efficient model optimization. The neural decoding framework was trained using the AdamW optimizer with a learning rate of $1 \times 10^{-4}$ and a weight decay parameter tuned via validation. We employed the GELU (Gaussian Error Linear Unit) activation function throughout the architecture to promote smooth and non-linear transformations in both the subject-specific alignment layers and the shared encoder. Our training was conducted for 10 epochs using mini-batches of size *N* (specified based on memory constraints per experiment). The contrastive objective was optimized using a temperature-scaled cosine similarity loss, as described in Section “Cross-subject and Cross-dataset Neural Vision Alignment”. The IP-Adapter image embeddings were extracted using a Stable Diffusion XL backbone, and all fMRI samples were aligned into a shared semantic space through the contrastive training pipeline. For reconstruction, ridge regression was applied to refine latent representations before passing them to the IP-Adapter+SDXL generative module. All hyperparameters were kept consistent across experiments to ensure fair evaluation across different subjects and datasets.

To further clarify the generalization setting and the contribution of individual components, we report two additional analyses. Table 5 presents the leave-one-dataset-out (LODO) transfer experiment, where the target dataset is excluded from source pretraining and used only for lightweight alignment-layer finetuning before test evaluation. Table 6 summarizes the main ablations for data augmentation, multi-dataset unified training, LODO transfer, and ridge refinement before SDXL reconstruction. These analyses complement the dataset-specific results in Tables 2–4 by separating unified multi-dataset training from stricter target-excluded transfer and by quantifying the effect of the main architectural and training components.

### Quantitative Results

We evaluate our proposed brain decoding framework across three benchmark datasets: NSD, BOLD5000, and GOD. To avoid ambiguity, we first define the five experimental protocols used throughout this section:

- **Within-dataset cross-subject** (e.g., *NSD*, *BOLD5000*, *GOD*): A single model is trained on all subjects from one dataset simultaneously, using a *pooled training* strategy. Each subject’s fMRI signals are routed through their own subject-specific alignment layer, while the shared Transformer encoder and contrastive objective are optimised jointly across all subjects.
- **Multi-dataset unified without finetuning** (e.g., *{Unified-NSD*, *Unified-BOLD5000*, *Unified-GOD*): A single unified model is trained on the training splits of NSD, BOLD5000, and GOD simultaneously. The target dataset is therefore included during training, but the target test split is held out. After training, the model is evaluated directly on each dataset’s test split, with no additional dataset-specific adaptation. This protocol evaluates whether one model can jointly support multiple heterogeneous datasets, but it should not be interpreted as a strict leave-one-dataset-out test.
- **Multi-dataset unified with alignment-layer finetuning** (e.g., *Unified-finetuned-BOLD5000*, *Unified-finetuned-GOD*): The multi-dataset unified model is frozen except for its subject-specific alignment layers, which are finetuned on the target dataset. This evaluates lightweight adaptation under limited data.
- **Leave-one-dataset-out (LODO) with target alignment-layer finetuning**: The target dataset is excluded during source pretraining. For example, in LODO-NSD the source model is pretrained only on BOLD5000 and GOD. The shared encoder is then frozen, and only the held-out target dataset’s subject-specific alignment layers and final alignment layer are finetuned using the target training split. Ridge refinement is also fit only on target training predictions before evaluating the target test split. This experiment tests transfer to a dataset not used during source pretraining, while still allowing lightweight target-domain adaptation.
- **Low-data transfer learning** (Fig. 6): The pretrained multi-dataset unified model’s alignment layers are finetuned using only a fraction (25%, 50%, 75%, or 100%) of the target dataset’s training data, and compared against within-dataset models trained from scratch on the same fractions.

To assess decoding performance, We adopt the standard evaluation protocol established by Brain-Diffuser (Ozcelik & VanRullen, 2023) and MindEye (Scotti et al., 2023), computing the following metrics between each reconstructed image and its corresponding ground-truth stimulus: **PixCorr** is the Pearson correlation between the flattened pixel intensities of the reconstructed and ground-truth images, after resizing both to *425 × 425* pixels and normalising to [0, 1]. It quantifies low-level structural correspondence. Higher is better (*↑*). **SSIM** (Structural Similarity Index) is computed on single-channel (grayscale) versions of the resized images at *425 × 425* resolution. It captures luminance, contrast, and structural similarity simultaneously. Higher is better (*↑*). **AlexNet(2) and AlexNet(5)** are the cosine similarities (reported as percentages, *× 100*) between intermediate feature activations at layers 2 and 5 of a pretrained AlexNet (Krizhevsky et al., 2012), extracted from reconstructed and ground-truth images. These capture low- to mid-level perceptual features. Higher is better (*↑*). **Inception** is the cosine similarity (*× 100*) between global average-pooled feature vectors from a pretrained Inception-v3 network, reflecting high-level semantic content. Higher is better (*↑*). **CLIP** is the cosine similarity (*× 100*) between image embeddings from a pretrained CLIP ViT-L/14 model (Radford et al., 2021), measuring vision-language semantic alignment. Higher is better (*↑*). **EfficientNet-B (EffNet-B)** and **SwAV** are distance-based retrieval metrics following the protocol of Brain-Diffuser (Ozcelik & VanRullen, 2023). For each test image, the pairwise distance between the reconstructed and ground-truth feature representations (EfficientNet-B0 and SwAV ResNet-50, respectively) is computed. Values are reported as the mean feature distance across the test set. Lower is better (*↓*). **Top-1 accuracy** is an embedding-based image retrieval metric. For each test sample, cosine similarities are computed between the predicted CLIP embedding and all ground-truth test embeddings; Top-1 accuracy is the fraction of samples for which the correct image ranks first. This differs from category classification: it is an instance-level retrieval task over the full test set. Higher is better (*↑*). Because the neural encoder is trained to predict IP-Adapter/OpenCLIP-like visual embeddings, CLIP-based metrics may be partially aligned with the training target and should not be interpreted in isolation. We therefore report complementary non-CLIP metrics, including pixel correlation, SSIM, AlexNet features, Inception features, EfficientNet-B distance, and SwAV distance. These metrics probe different levels of reconstruction fidelity and help separate semantic alignment from low-level spatial accuracy.

**Fig. 2:**
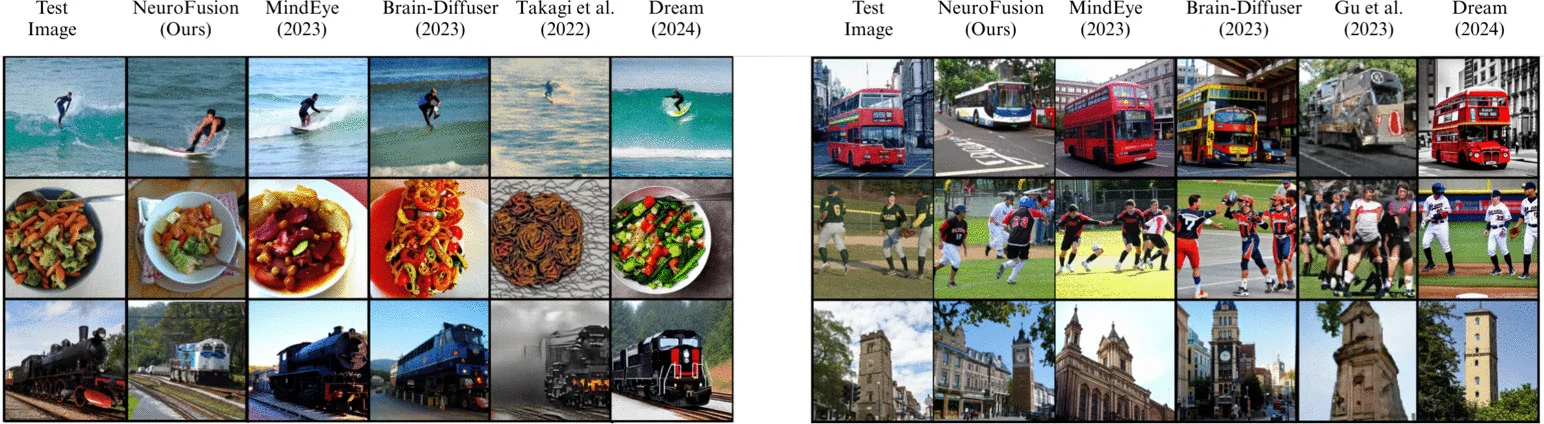
Qualitative comparison of reconstructed images from the NSD dataset. Each row presents a test image (leftmost column) alongside reconstructions. The reconstructions are evaluated for both perceptual similarity and semantic fidelity, illustrating the ability of each method to capture visual details and high-level content from brain activity

**Fig. 3:**
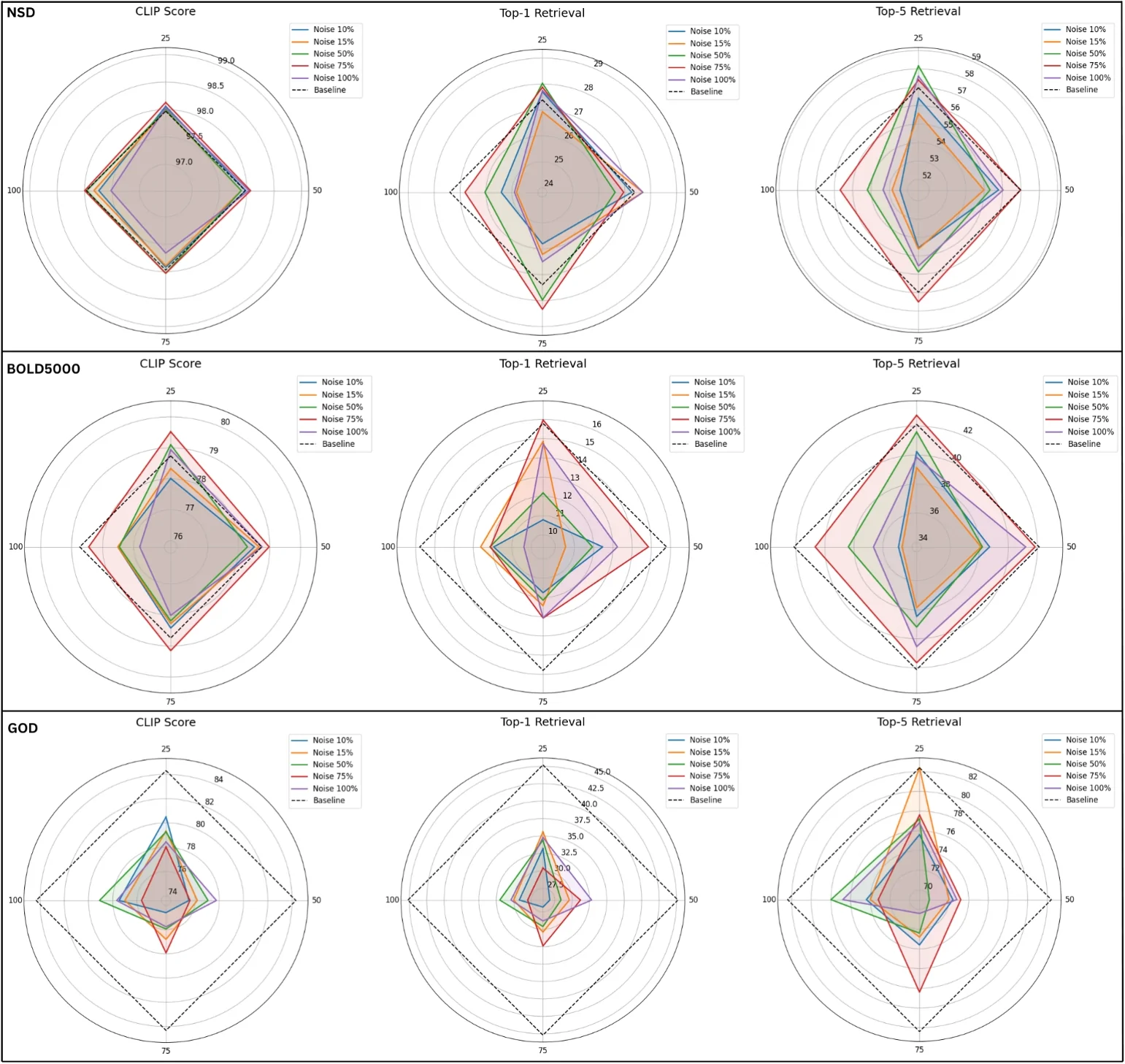
Quantitative evaluation of the data augmentation framework. Each radar plot summarizes the effects of varying augmentation percentages (25%, 50%, 75%, 100%) and noise scales (10, 15, 50, 75, 100) on three key metrics: CLIP Score, Top-1 Retrieval, and Top-5 Retrieval. For each configuration, synthetic fMRI responses were generated by projecting novel image embeddings into subject-specific neural space using ridge regression, followed by controlled noise perturbation sampled from empirical decoding residuals

**Fig. 4:**
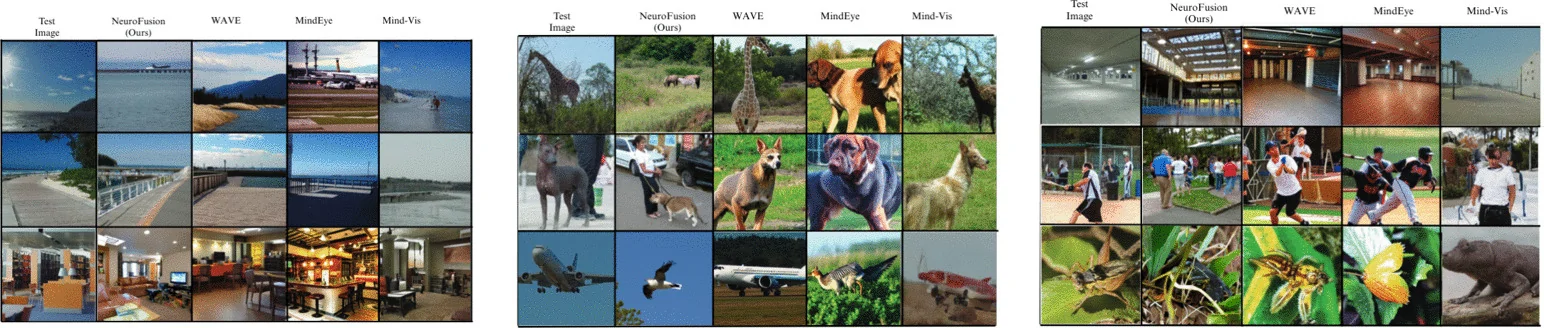
Qualitative comparison of reconstructed images from the BOLD5000 dataset. Each row displays a test image (leftmost column) and corresponding reconstructions. The reconstructions are evaluated for both perceptual similarity and semantic fidelity, illustrating the ability of each method to capture visual details and high-level content from brain activity

**Fig. 5:**
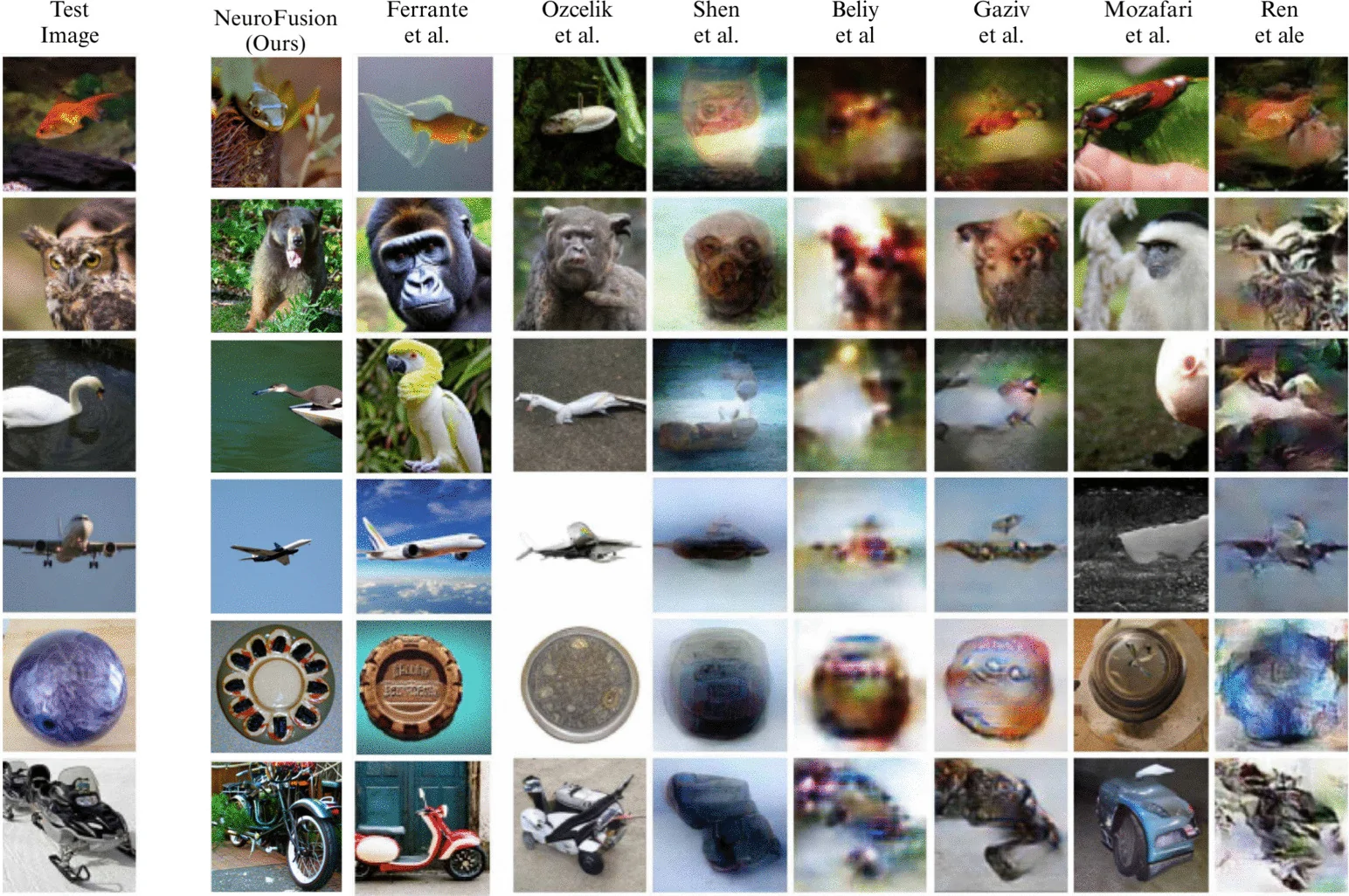
Qualitative comparison of reconstructed images from the GOD dataset. Each row displays a test image (leftmost column) and corresponding reconstructions. The reconstructions are evaluated for both perceptual similarity and semantic fidelity, illustrating the ability of each method to capture visual details and high-level content from brain activity

**Fig. 6:**
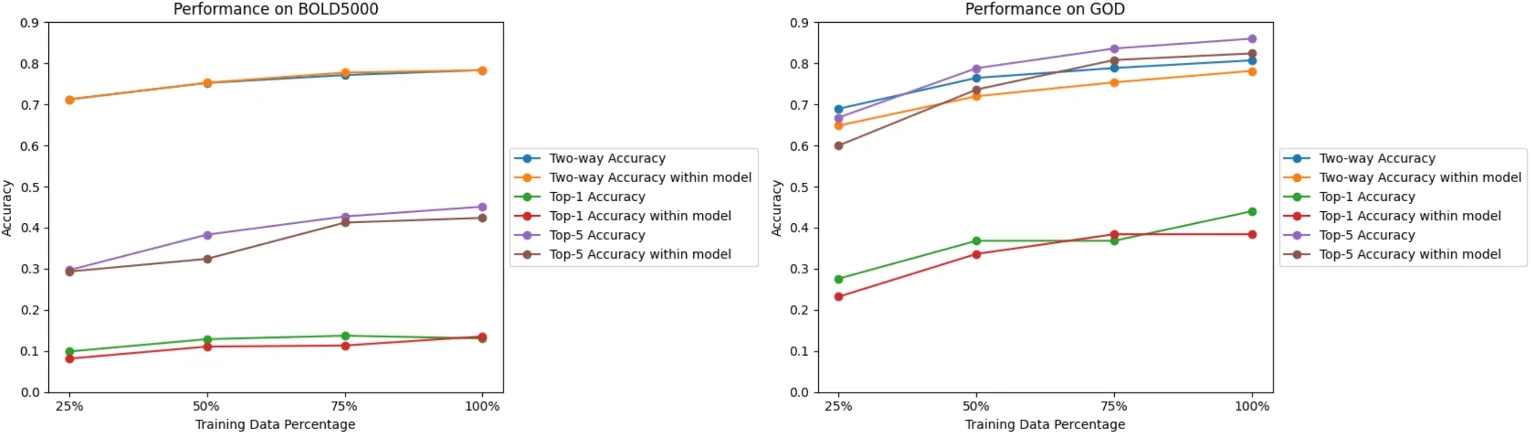
Comparison of model performance on the BOLD5000 (left) and GOD (right) datasets across varying proportions of training data under two training paradigms. In setting I ”within-model” approach, models were trained from scratch on each dataset using different amounts of data (cross-subject evaluation). In setting II, a pretrained model (on NSD, BOLD5000, and GOD) was used, with all parameters frozen except the alignment layers, which were finetuned on the target dataset. Results indicate that higher training data percentages lead to improved accuracy, and that finetuning alignment layers enables effective transfer to new datasets with limited data

To quantify the impact of our data augmentation strategy, we provide decoding results under both augmented (AUG) and non-augmented training settings across Natural scenes dataset (NSD) and BOLD5000 datasets (see Tables 2 and 3). Consistently, models trained with augmentation outperform their non-augmented counterparts across multiple metrics, including SSIM, CLIP similarity, and retrieval accuracy. For example, on the NSD dataset, our ”NSD (AUG)” variant improves CLIP similarity from 93% to 94.8% and Inception similarity from 91.7% to 94.1%, while SSIM increases from 0.328 to 0.346. This trend holds across BOLD5000 as well, confirming that our augmentation approach enhances the semantic alignment. These gains are especially prominent in cross-dataset transfer scenarios, where data scarcity and distributional shift typically impair performance, highlighting the role of augmentation in improving generalization. Table 2 summarizes the reconstruction quality on the NSD dataset. The upper section reports prior cross-subject baselines, while the lower section includes our models: the “NSD” model trained and evaluated on NSD (cross-subject) and the “Unified-NSD” variant, which is trained jointly on the training splits of NSD, BOLD5000, and GOD and tested on the held-out NSD test split. Compared to strong baselines such as MindReader (Lin et al., 2022), Gu et al. (2022), Brain-Diffuser (Ozcelik & VanRullen, 2023), MindEye (Scotti et al., 2023), and Dream (Xia et al., 2024), our models achieve highly competitive performance. Our approach is most competitive on semantic metrics (e.g., CLIP/Inception similarities), consistent with optimizing alignment in a semantic embedding space; low-level structural metrics (PixCorr) are lower than methods explicitly optimized for pixel-level fidelity. Notably, our “NSD” variant achieves the highest CLIP similarity (94.8%) and Inception similarity (94.1%), even outperforming several models optimized specifically for within-dataset training. Although PixCorr values are lower compared to MindEye, this is an expected outcome given our model’s reliance on IP-Adapter embeddings, which emphasize semantic alignment over low-level structural detail. This reflects a deliberate design choice that prioritizes perceptual fidelity in the latent space over pixel-wise accuracy. Table 3 reports results on the BOLD5000 dataset. Our “Unified-BOLD5000” variant (trained jointly across datasets and directly evaluated on the held-out BOLD5000 test split) demonstrates strong multi-dataset performance, surpassing prior cross-subject baselines such as Mind-Vis (Chen et al., 2023) and WAVE (Wang et al., 2024b) in several semantic metrics. For instance, it achieves 68.5% and 69.9% similarity in AlexNet(2) and Inception respectively, with 19.6% top-1 retrieval accuracy. After finetuning only the subject-specific alignment layers (“Unified-finetuned-BOLD5000”), the SSIM improves to 0.403—matching the highest reported SSIM in the literature—demonstrating that lightweight adaptation is sufficient to recover fine-grained visual detail. This property is especially important for practical deployment in scenarios with limited subject-specific data. Table 4 presents results on the GOD dataset. Despite the increased difficulty posed by this dataset—due to differences in stimulus type, experimental protocol, and subject pool—our framework maintains high semantic decoding performance. The “Unified-GOD” variant achieves 81.0% CLIP similarity and 68.3% Inception similarity without any finetuning. After tuning the alignment layers (“Unified-finetuned-GOD”), the model retains robust performance across all metrics, with improvements in SSIM and stability in semantic alignment. Table 5 further evaluates the stricter LODO setting. In this setting, the target dataset is absent from source pretraining and enters only during target alignment-layer finetuning; the resulting performance remains below the strongest within/unified settings on some metrics but confirms that the learned shared encoder can be adapted to a previously excluded dataset without retraining the full model. Table 6 summarizes the component ablations. The ridge-refinement ablation shows that refinement substantially improves CLIP similarity and Top-1 retrieval across representative settings, although SSIM can be mixed because the refinement is optimized in embedding space rather than directly for pixel-level structure. These results suggest that shared visual representations learned from multiple datasets can generalize across both anatomical and acquisition-level variability. Qualitative results in Figs. 2, 4, and in Fig. 5 illustrate that the reconstructions generated from fMRI signals across subjects and datasets preserve high-level semantic content, including object categories, scene structure, and visual themes. This semantic plausibility should not be equated with pixel-faithful reconstruction: diffusion priors can recover plausible object and scene content while altering fine spatial details. While structural details may vary due to dataset-specific factors and our semantic embedding space, perceptual similarity remains consistent. We further evaluate our subject-aware data augmentation strategy Fig. 3, which shows that augmenting training data using ridge-regression-generated neural signals with sampled noise improves decoding metrics across multiple conditions. Figure 6 provides additional insights into the adaptability of our proposed framework in low-data regimes. We compare two training paradigms: (I) a “within-model” baseline where models are trained from scratch on each dataset using different proportions of training data, and (II) our transfer learning approach, where a pretrained model—trained across NSD, BOLD5000, and GOD—is frozen except for subject-specific alignment layers, which are finetuned using limited data from the target dataset. The results clearly show that even with reduced data availability (as low as 25%), finetuning only the alignment layers yields comparable or superior decoding performance to fully-trained models. This finding is especially significant for real-world settings, such as clinical neuroimaging or brain-computer interface applications, where collecting large-scale fMRI data is impractical. These results validate that the shared semantic latent space learned during cross-dataset pretraining is transferable, and that lightweight adaptation is sufficient to generalize to new domains. In summary, Fig. 6 empirically confirms the efficiency, robustness, and scalability of our modular training strategy under practical constraints.

**Table 2:** Quantitative comparison of image reconstruction on the NSD dataset using structural (low-level) and semantic (high-level) metrics. Results above the horizontal line are from prior cross-subject studies. Below are our results: “NSD” refers to models trained and evaluated on NSD (cross-subject), while “Unified-NSD” denotes the multi-dataset unified model trained jointly on the training splits of NSD, BOLD5000, and GOD and evaluated on the held-out NSD test split. The “(AUG)” variants incorporate data augmentation during training, demonstrating improved performance across both low- and high-level metrics

Method	PixCorr *↑*	SSIM *↑*	AlexNet(2) *↑*	AlexNet(5) *↑*	Inception *↑*	CLIP *↑*	EffNet-B *↓*	SwAV *↓*	Top-1 acc *↑*
Mind-Reader (Lin et al., 2022)	–	–	–	–	78.2%	–	–	–	–
Takagi and Nishimoto (2023)	–	–	83.0%	83.0%	76.0%	77.0%	–	–	–
Gu et al. (2022)	.150	.325	–	–	–	–	.862	.465	–
Brain-Diffuser (Ozcelik & VanRullen, 2023)	.254	.356	94.2%	96.2%	87.2%	91.5%	.775	.423	–
MindEye (Scotti et al., 2023)	**.309**	.323	**94.7%**	**97.8%**	**93.8%**	**94.1%**	.645	.367	–
Dream (Xia et al., 2024)	.274	.328	93.9%	96.7%	93.4%	94.1%	.645	.418	–
NSD	.071	.328	80.5%	91.2%	91.7%	93.0%	.707	.398	55.8%
NSD (AUG)	.077	.346	83.6%	93.0%	**94.1%**	**94.8%**	.675	.383	61.1%
Unified-NSD	.069	.326	79.7%	90.8%	91.5%	92.9%	.711	.401	55.5%
Unified-NSD (AUG)	.064	.337	80.3%	91.5%	92.2%	93.5%	.698	.392	57.8%

**Table 3:** Quantitative evaluation of image reconstruction on the BOLD5000 dataset using low-level (structural) and high-level (semantic) metrics. Results above the horizontal line are from prior cross-subject studies. Below the line are our results: “BOLD5000” indicates cross-subject models trained and evaluated on BOLD5000; “Unified-BOLD5000” denotes the multi-dataset unified model trained jointly on the training splits of NSD, BOLD5000, and GOD and evaluated on the held-out BOLD5000 test split; and “Unified-finetuned-BOLD5000” represents the same unified model after finetuning only the BOLD5000 alignment layers. The “(AUG)” variants use data augmentation during training, leading to notable improvements in reconstruction quality across most metrics

Method	PixCorr *↑*	SSIM *↑*	AlexNet(2) *↑*	AlexNet(5) *↑*	Inception *↑*	CLIP *↑*	EffNet-B *↓*	SwAV *↓*	Top-1 acc *↑*
WAVE (Wang et al., 2024b)	**.050**	0.194	**69.47%**	**78.31%**	**68.41%**	**78.41%**	.902	.591	**20.75%**
Mind-Vis (Chen et al., 2023)	.036	**.272**	60.56%	68.92%	64.25%	64.47%	**.938**	**.593**	9.19%
BOLD5000	.036	.374	58.9%	64.0%	60.2%	62.2%	.947	.652	10.2%
BOLD5000 (AUG)	.033	.403	65.5%	**78.4%**	**69.2%**	75.7%	**.900**	**.549**	19.1%
Unified-BOLD5000	.037	.397	61.5%	71.0%	64.4%	71.1%	.924	.580	13.4%
Unified-BOLD5000 (AUG)	.034	.375	68.5%	**79.0%**	**69.9%**	75.4%	**.911**	**.557**	19.6%
Unified-finetuned-BOLD5000	.040	.403	65.9%	76.2%	69.5%	76.0%	.904	.553	19.3%

**Table 4:** Quantitative evaluation of image reconstruction on the GOD dataset using structural (low-level) and semantic (high-level) metrics. Results above the horizontal line are from prior cross-subject studies. Below the line are our results: “GOD” denotes cross-subject models trained and evaluated on GOD; “Unified-GOD” indicates the multi-dataset unified model trained jointly on the training splits of NSD, BOLD5000, and GOD and evaluated on the held-out GOD test split; and “Unified-finetuned-GOD” represents the same unified model after finetuning only the GOD alignment layers

Method	PixCorr *↑*	SSIM *↑*	AlexNet(2) *↑*	AlexNet(5) *↑*	Inception *↑*	CLIP *↑*	EffNet-B *↓*	SwAV *↓*	Top-1 acc *↑*
Koide-Majima et al. (2024)	-	-	-	-	-	90.00%	-	-	-
Ferrante et al. (2024b)	.32	.38	67.00%	68.00%	66.00%	69.00%	-	-	-
GOD	.041	.331	**79.1%**	**88.5%**	**73.2%**	84.0%	.884	.532	19.2%
Unified-GOD	.028	.337	**72.3%**	**84.9%**	**68.3%**	81.0%	.913	.561	16.1%
Unified-finetuned-GOD	.033	.313	**69.1%**	**82.4%**	**68.5%**	79.8%	.916	.562	15.4%

**Table 5:** Leave-one-dataset-out (LODO) transfer results with target alignment-layer finetuning. For each target dataset, source pretraining excludes the target dataset entirely. The shared encoder is then frozen, only the target subject-specific alignment layers and final alignment layer are finetuned using the target training split, and ridge refinement is fit on target training predictions before evaluation on the held-out target test split

Target dataset	PixCorr *↑*	SSIM *↑*	AlexNet(2) *↑*	AlexNet(5) *↑*	Inception *↑*	CLIP *↑*	EffNet-B *↓*	SwAV *↓*	Top-1 acc *↑*
LODO-NSD + alignment FT	.064	.332	79.5%	90.7%	91.6%	93.2%	.710	.400	56.4%
LODO-BOLD5000 + alignment FT	.023	.401	64.6%	73.3%	66.9%	72.9%	.919	.564	16.5%
LODO-GOD + alignment FT	.038	.305	75.1%	86.6%	71.5%	79.2%	.896	.560	19.9%

**Table 6:** Ablation summary for the main framework components. Values follow the same point-estimate format as the main result tables. The ridge-refinement rows compare SDXL reconstruction from the raw predicted embedding against reconstruction from the ridge-refined embedding

Component tested	Comparison	SSIM *↑*	CLIP *↑*	Top-1 acc *↑*
Data augmentation	NSD *→* NSD (AUG)	.328 *→* .346	93.0% *→* 94.8%	55.8% *→* 61.1%
Data augmentation	BOLD5000 *→* BOLD5000 (AUG)	.374 *→* .403	62.2% *→* 75.7%	10.2% *→* 19.1%
Unified training	BOLD5000 (AUG) *→* Unified-BOLD5000 (AUG)	.403 *→* .375	75.7% *→* 75.4%	19.1% *→* 19.6%
LODO transfer	Unified-BOLD5000 *→* LODO-BOLD5000 + alignment FT	.397 *→* .401	71.1% *→* 72.9%	13.4% *→* 16.5%
Ridge refinement	NSD (AUG), raw *→* refined	.315 *→* .346	80.9% *→* 94.8%	37.9% *→* 61.1%
Ridge refinement	BOLD5000 (AUG), raw *→* refined	.398 *→* .403	66.4% *→* 75.7%	14.0% *→* 19.1%
Ridge refinement	Unified-BOLD5000 (AUG), raw *→* refined	.391 *→* .375	67.0% *→* 75.4%	12.9% *→* 19.6%

## Discussion

Our study demonstrates that cross-subject and cross-dataset brain decoding is not only feasible but can also achieve competitive and in some cases SOTA performance under realistic and challenging conditions. Unlike traditional approaches, which typically train separate models for each subject or dataset, our model generalizes across diverse datasets (NSD, BOLD5000, GOD) and cohorts using a unified architecture, thereby enabling broader applicability and real-world scalability. Importantly, our framework is trained on what is, to our knowledge, the largest combined dataset for brain-to-vision modeling to date, spanning approximately 42,584 unique visual stimuli, 13 subjects, and *∼*72.5 hours of functional fMRI acquisition time (computed as total trials *×* TR per dataset; see Table 7). This scale enables training of high-capacity models with improved generalization, and establishes a foundation for future neural foundation models of vision. Several key insights emerge from our results. First, we observe strong performance from our multi-dataset unified model on held-out test splits, particularly on high-level semantic metrics. The LODO experiment provides a stricter complementary test, showing that a source-pretrained shared encoder can be adapted to a previously excluded dataset through lightweight target alignment-layer finetuning. This outcome highlights the efficacy of aligning brain signals with pretrained image embeddings from IP-Adapter, which are designed to encode semantic attributes of the visual stimuli. Given that the IP-Adapter prioritizes semantic consistency over low-level structural fidelity, our reconstruction pipeline focuses on capturing category-level and perceptual meaning, rather than fine-grained pixel arrangements. Consequently, structural similarity scores (e.g., SSIM, PixCorr) may be lower compared to models that explicitly optimize for spatial detail; we interpret this as an explicit trade-off and limitation of the current embedding-driven design, rather than as evidence of uniformly superior reconstruction quality. Because reconstructions are generated under a strong diffusion prior, outputs may exhibit hallucinated details that improve semantic metrics without reflecting voxel-level fidelity. For this reason, CLIP-based scores are treated as semantic-alignment evidence and are interpreted together with non-CLIP metrics such as PixCorr, SSIM, AlexNet, Inception, EfficientNet-B, and SwAV. We therefore interpret semantic similarity metrics as evidence of aligned high-level content rather than pixel-faithful recovery, and we recommend complementary analyses (e.g., retrieval against large candidate sets, ROI ablations, and calibration of uncertainty).

Future work could complement this semantic decoding with structural priors if finer detail is required for specific applications. Second, we show that finetuning only the subject-specific alignment layers of a pretrained model significantly boosts performance on novel datasets, especially in low-data regimes. As illustrated in Fig. 6, our two-stage training setup enables effective transfer: models trained from scratch with more training samples yield expected improvements, but even when using limited data, finetuning the alignment layers alone allows the pretrained model to adapt quickly to new subjects and datasets. This finding underscores the modularity and efficiency of our design and opens avenues for practical deployment where large-scale retraining is not feasible. Third, we introduce a data augmentation strategy that leverages ridge regression to generate synthetic brain responses to novel images. By simulating plausible fMRI activity using pretrained decoders and injecting noise sampled from empirical residual distributions, we extend the diversity and scale of training data without requiring new neuroimaging scans. Figure 3 demonstrates that such augmentation consistently improves decoding performance, particularly under constrained training scenarios. However, the observed improvements are modest in some settings. This may reflect inherent limitations in the augmentation process: current generative models may not fully capture the nuances of neural activity, and variability in real brain responses could stem from factors not modeled by our approach such as attentional state, trial history, or individual-specific encoding. These challenges have also been observed in prior work (Piskovskyi et al., 2024), and addressing them may require more biologically grounded simulation methods or hybrid generative-neural decoders.

Our findings demonstrate the efficacy of cross-subject and cross-dataset alignment strategies in brain decoding, particularly when leveraging shared latent representations. Recent advancements have shown that such alignment is achievable through contrastive learning frameworks that match neural activity with image or language embeddings in a shared space (Sun et al., 2023; Wang et al., 2024a; Xia et al., 2024). These approaches reduce subject-specific variance and facilitate generalization across individuals and datasets. In our case, the use of subject specific alignment layers and joint training objectives led to robust decoding performance across domains, consistent with recent reports highlighting the benefits of aligning fMRI responses with foundation model embeddings (Rajabi et al., 2025; Zhang et al., 2025). Importantly, these results support the practical learnability of shared semantic mappings across datasets, but they do not prove that different brains share an identical representational geometry. Part of the transfer may be supported by the structure of the pretrained visual embedding space, the diffusion prior, shared natural-image statistics, and the capacity of the alignment layers to absorb subject- and dataset-specific differences.

### Limitations

Despite our framework’s scalability, several limitations remain. First, although our dataset is large relative to prior brain decoding studies, it still comprises only **13 subjects**, which is orders of magnitude smaller than typical vision-language datasets. Second, while our data augmentation method is principled, its effectiveness is constrained by the assumptions of linear ridge regression and the quality of residual noise modeling. Future directions could involve more expressive generative models or reinforcement from real neural variability. Third, the current model prioritizes semantic alignment at the expense of structural detail, which may not suit applications requiring fine-grained visual reconstructions. Fourth, because the model target is an OpenCLIP/IP-Adapter embedding, evaluation metrics from related semantic embedding families can be favorable to the proposed method; this motivates reporting non-CLIP metrics and developing additional human or task-based evaluations in future work. Finally, ethical considerations surrounding brain decoding must be addressed particularly regarding bias amplification, potential misuse, and implications for privacy when scaling BCI technologies.

### Broader Impact

Our work contributes toward the long term goal of neural foundation models of vision that generalize across subjects and tasks. This framework opens avenues for in-silico experiments using encoding models, and enables analysis of residual activity in shared embedding spaces to study cognitive differences across individuals. As we advance brain-to-vision translation, such capabilities may support future applications in clinical neuroscience, cognitive modeling, and human-computer interaction.

**Table 7:** Scale of the combined training corpus used in this study. Scan time is computed as total fMRI trials *×* TR for each dataset, representing functional acquisition time excluding inter-run breaks and setup. Unique stimuli for NSD are estimated as $4 \times 8{,}859$ subject-unique training images *+ 982* shared test images; BOLD5000 and GOD stimuli are shared across subjects

Dataset	Subjects used	TR (s)	Unique stimuli	Train trials	Test trials	Scan time (h)
NSD (Allen et al., 2022)	4 (of 8)	1.6	*∼*36,418	99,920	11,080	49.3
BOLD5000 (Chang et al., 2019)	4 (of 4)	2.0	4,916	19,212	452	10.9
GOD (Horikawa & Kamitani, 2017)	5 (of 5)	3.0	1,250	6,000	8,750	12.3
**Total**	**13**	–	*∼***42,584**	**125,132**	**20,282**	*∼***72.5**

## Conclusion

We presented a generalized brain decoding framework that reconstructs visual stimuli from fMRI signals across multiple datasets and subjects. By aligning neural activity with visual embeddings from a vision-language model (IP-Adapter+SDXL) via contrastive learning and introducing synthetic fMRI augmentation, we tackled key challenges in cross-subject and cross-dataset generalization. Experiments show that a unified model can decode robustly across heterogeneous datasets with minimal performance loss, narrowing the gap between subject-specific and generalized approaches. Additionally, lightweight finetuning of alignment layers enables rapid adaptation to new subjects or datasets, addressing data scarcity in brain-computer interface applications. While our approach improves generalization, low-level structural fidelity still lags in diverse settings. Future work will explore advanced domain adaptation, individualized cortical alignment, and multimodal learning to enhance decoding performance. Overall, our framework offers a scalable path toward universal brain-computer interfaces and advances the practical impact of neural decoding research.

## Information Sharing Statement

This study uses only publicly available, previously collected fMRI datasets. No new human participant data were collected for this work. The Natural Scenes Dataset (NSD) version 1.0 is available at https://naturalscenesdataset.org/; BOLD5000 is available at https://bold5000-dataset.github.io/website/; and the Generic Object Decoding (GOD) dataset version 1.2.1 is available through OpenNeuro at https://openneuro.org/datasets/ds001246/versions/1.2.1. Use of these datasets is subject to the access conditions and licenses specified by the original dataset providers.The analysis and model code used in this study has been made available in an anonymous GitHub repository: https://github.com/r8832711/Cross-subject-and-cross-dataset-brain-decoding. Pretrained models used in the reconstruction pipeline, including Stable Diffusion XL, IP-Adapter, and OpenCLIP components, are available from their respective public repositories and are cited in the manuscript. Derived outputs, including additional reconstruction examples and trained model checkpoints, can be made available by the corresponding author upon reasonable request, subject to the licensing and redistribution constraints of the original datasets and pretrained models.

## Data Availability

The NSD (Natural Scenes Dataset) version 1.0 used in this study is publicly available at https://naturalscenesdataset.org/. The dataset is released under the Creative Commons Attribution-NonCommercial (CC BY-NC 4.0) license. The BOLD5000 dataset used in this study is publicly available at https://bold5000-dataset.github.io/website/ and is released under the Creative Commons Attribution-NonCommercial-ShareAlike (CC BY-NC-SA 4.0) license. The GOD (Generic Object Decoding) dataset version 1.2.1 used in this study is available from OpenNeuro at https://openneuro.org/datasets/ds001246/versions/1.2.1 and is released under the Public Domain Dedication and License (PDDL). The code used in this study has been anonymously uploaded to GitHub and is available at: https://github.com/r8832711/Cross-subject-and-cross-dataset-brain-decoding
